# A novel role for bone marrow-derived cells to recover damaged keratinocytes from radiation-induced injury

**DOI:** 10.1038/s41598-021-84818-1

**Published:** 2021-03-11

**Authors:** Junko Okano, Yuki Nakae, Takahiko Nakagawa, Miwako Katagi, Tomoya Terashima, Daisuke Nagakubo, Takashi Nakayama, Osamu Yoshie, Yoshihisa Suzuki, Hideto Kojima

**Affiliations:** 1 Department of Plastic and Reconstructive Surgery, Shiga University of Medical Science, Shiga, Japan; 2 Department of Stem Cell Biology and Regenerative Medicine, Shiga University of Medical Science, Shiga, Japan; 3 Department of Nephrology, Otowa Hospital, Kyoto, Japan; 4 Faculty of Pharmaceutical Sciences, Division of Health and Hygienic Sciences, Himeji Dokkyo University, Hyogo, Japan; 5 Division of Chemotherapy, Faculty of Pharmacy, Kindai University, Osaka, Japan; 6 The Health and Kampo Institute, Miyagi, Japan

**Keywords:** Biophysics, Biotechnology, Cell biology, Developmental biology

## Abstract

Exposure to moderate doses of ionizing radiation (IR), which is sufficient for causing skin injury, can occur during radiation therapy as well as in radiation accidents. Radiation-induced skin injury occasionally recovers, although its underlying mechanism remains unclear. Moderate-dose IR is frequently utilized for bone marrow transplantation in mice; therefore, this mouse model can help understand the mechanism. We had previously reported that bone marrow-derived cells (BMDCs) migrate to the epidermis-dermis junction in response to IR, although their role remains unknown. Here, we investigated the role of BMDCs in radiation-induced skin injury in BMT mice and observed that BMDCs contributed to skin recovery after IR-induced barrier dysfunction. One of the important mechanisms involved the action of CCL17 secreted by BMDCs on irradiated basal cells, leading to accelerated proliferation and recovery of apoptosis caused by IR. Our findings suggest that BMDCs are key players in IR-induced skin injury recovery.

## Introduction

Exposure of the skin to moderate dose (1–10 Gy) of ionising radiation (IR) occurs during radiation therapy against cancer^1^. However, accidental exposure to X- and γ-radiation also occurs in modern medical society^2^. In the past, incorrect estimation of doses by unskilled staff, old version of computer files used for calculating treatment time, or incorrect repair of accelerators resulted in patients receiving overdoses of radiation^3^. Nowadays, owing to the increase in the population of elderly patients with cancer due to extended life expectancy, the frequency of advanced radiotherapies, such as cyberknife for eradicating metastatic cancers, which are usually incurable using surgical treatments, is increasing^4^. Generally, IR exposure to the skin can cause erythema, edema, or desquamation in mild cases in several hours or days, and intractable ulcers in decades^2^. However, the mechanisms underlying radiation-induced skin injury remain elusive, and the details regarding radiation accumulation after cyberknife treatment that cause skin injury are negligible.

As reports on humans are limited, studies using relevant animal models help investigate mechanisms and design novel therapies for radiation-induced skin injury. Moderate-dose IR is often utilized in laboratories for eliminating recipient bone marrow cells for bone marrow transplantation (BMT). Thus, a BMT mouse model will be an ideal tool for investigating the role of bone marrow-derived cells in radiation-induced skin injury. Recently, we have demonstrated that 10 Gy IR causes mild inflammation in the skin where bone marrow-derived cells (BMDCs) migrate into the epidermis-dermis junction (EDJ) in BMT mice^5^. In this study, we attempted to investigate the roles of BMDCs in IR-induced skin injury further and demonstrated that BMDCs in EDJ were novel components for the damaged epidermal cell recovery from a disrupted skin barrier. Our findings will be useful for the development of regenerative therapy for patients suffering from incurable skin ulcers caused by IR.

## Results

### BMT blocked IR-induced skin barrier disruption

**Figure 1:**
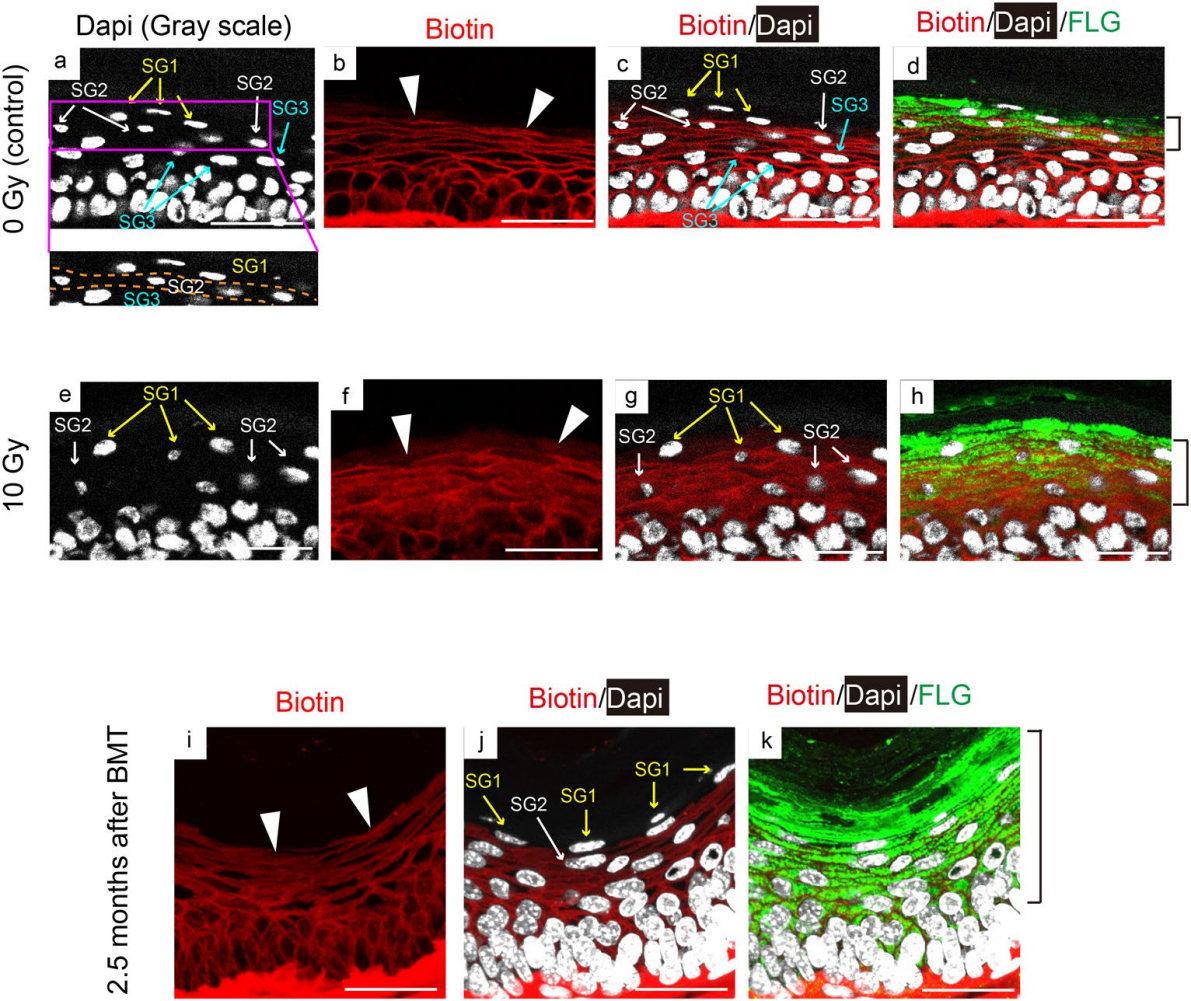
BMDCs block IR-induced skin barrier defects. Histological images of the paw pad skin of control (**a**–**d**) and 10 Gy- (**e**–**h**) irradiated mice 7 days after IR are shown. High-power view (**a**) shows the alignment of granular layers (SG1-3) using orange dotted lines. Biotin tracer (red) injected dermally is visualized with DAPI (greyscale) and staining with anti-filaggrin (FLG; green). (**i**–**k**) Biotin staining and merged staining (biotin and filaggrin with DAPI) of the paw pad skin of mice receiving allogeneic bone marrow transplantation (BMT) after 10 Gy irradiation. The skin 2.5 months after BMT was shown. Arrowheads indicate individual biotin stain patterns, while brackets show the filaggrin-positive layers. Scale bars, 20 µm. IR, ionising radiation; BMDCs, bone marrow-derived cells; BMT, bone marrow transplant.

First, the effect of irradiation on the skin barrier was morphologically examined in 10 Gy irradiated mice using the biotin function assay. Physiologically, the granular layers of mouse skin (SG) are composed of three layers (SG1, 2, and 3). Epidermal cells differentiate, and move up from SG3 to SG1 layers, though the tight junction is located in the SG2 layer. Biotin is a small molecule that usually diffuses to the epidermal layer. Usually, biotin is diffusely spread but is restricted below SG2, whereas it spreads to SG1 in case the tight junction is impaired due to skin barrier dysfunction^6^. We observed that injected biotin subcutaneously spread into intercellular spaces up to only SG2 in normal skin (Fig. 1a–c); however, 10 Gy irradiation changed this pattern. While it did not change on day 5 (Supplemental Fig. 1), biotin spread around keratinocytes in the SG1 layer on day 7, indicating that the skin barrier function was impaired (Fig. 1e–g and compare arrowheads in b and f). Next, we performed staining using anti-filaggrin antibody, along with the biotin assay, to further evaluate the differentiation layers^7^ (Fig. 1d, bracket). The differentiation layers appeared to be thicker than the control layer 7 days after exposure to 10 Gy, consistent with radiation dermatitis^8^ (brackets in Fig. 1d, h). Furthermore, the nucleus of keratinocytes in the SG1 and SG2 layers showed oval shape after 7 days, which was one of the characteristics observed in apoptotic cells^9^, although they appeared spindle-like in the control skin (Fig. 1a, e). To further investigate the effects of radiation on the skin, we attempted to isolate keratinocytes (basal and suprabasal cells) from epidermal layers. The mechanical peeling of epidermal layers from the dermis, followed by enzymatic digestion of the EDJ, is a useful way for isolating keratinocytes^5,10^. Using this method, we sorted keratinocytes as CD45^−^/MHCII^−^ cells from the epidermal cell suspension of mice 7 days after 10 Gy IR exposure or from age-matched control to exclude CD45^+^ and/or MHCII^+^ cells. Apoptotic cells were then identified as annexin V^+^ amine-reactive dye^+^ population^11^. As a result, we observed that the number of apoptotic keratinocytes was significantly higher in 10 Gy irradiated mice than in control mice, indicating the harmful effects of 10 Gy irradiation in the skin at that point (Supplemental Fig. 2).

**Figure 2:**
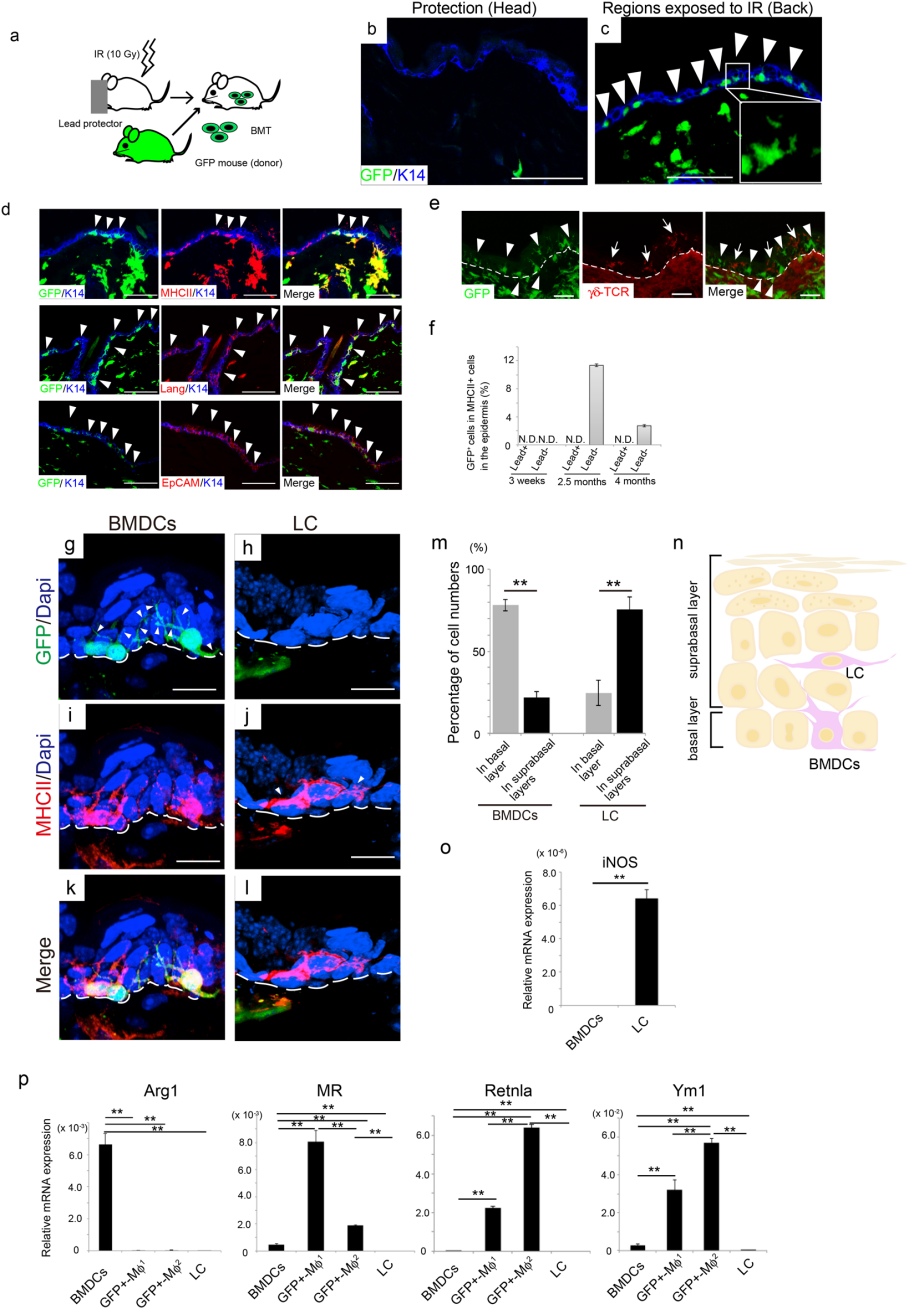
BMDCs that migrated into EDJ in response to IR are distinct from LC and macrophages. (**a**) Schema of establishing BMT mice with head protectors against irradiation. After irradiation in such a way, 4 × 10^6^ bone marrow cells isolated form green fluorescent protein (GFP)-tg mice were injected in tail vein of the recipient mice. (**b**, **c**) BMDCs were not observed in the epidermis of the protected area, while they were observed in the unprotected area. Arrowheads indicated GFP-positive cells in the basal layer of the skin. Keratin 14 (K14; blue) marked the basal layer of the skin. High magnification showed ramified GFP-positive cells. (**d**) Co-localization of various markers with the GFP-positive cells. MHCII, major histocompatibility complex II; Lang, langerin; EpCAM, epithelial cell adhesion molecule. Arrowheads indicate co-localisation of the markers with GFP-positive cells in the basal layer. (**e**) γδ-TCR, a marker of dendritic epidermal T cells, did not co-localise with GFP-positive cells. Arrowheads and arrows indicate GFP-positive cells and γδ-TCR-positive cells, respectively. Dotted lines show EDJ. Scale bars, 50 µm. (**f**) Percentage of GFP-positive cells in MHCII-positive cells in the epidermis of unprotected (Lead^−^) and protected (Lead^+^) areas. N.D., not detected. Bars are shown as mean ± SE. Three mice were examined. (**g**–**l**) MHCII immunostaining in BMDCs and LCs. Dotted lines show EDJ. Arrowheads show ramifications of BMDCs or LCs. Scale bars, 10 µm. (**m**) Quantitative analyses of the distribution of BMDCs and LCs in the layers of the epidermis. The distribution of BMDCs and LC in skin layers was examined in approximately 90 cells. Three mice were examined. (**n**) Schema of (**g**–**m**). (**o**) Nitric oxide synthase (iNOS) was significantly expressed in LC, but not in BMDCs. (**p**) Comparison of mRNA expression of macrophage markers among BMDCs, Mφ^1^, Mφ^2^, and LC. Bars show expression relative to a housekeeping gene (RPLP0) ± SE. **P < 0.01. EDJ, epidermis-dermis junction; BMDCs, bone marrow-derived cells; LC, Langerhans cells.

“Next, we examined the skin of the recipient BMT mice receiving allogeneic BMT after 10 Gy irradiation. The biotin function assay showed that the skin of these mice after 2.5 months BMT exhibited reticular patterns of biotin, which was identical to those in control mice (Fig. 1i, compare with 1b). Moreover, the nuclei of keratinocytes in the SG1 and SG2 layers turned out to be morphologically spindle-like, similar to those of skin under control conditions (Fig. 1j, compare with 1c). Considering that morphology of nuclei appears apoptotic and that barrier functions were damaged in the 10 Gy irradiated skin after 10 days, these results strongly indicated that the irradiated skin followed by BMT was recovered morphologically and functionally in 2.5 months.

The filaggrin-positive layers were still thicker in BMT mice than in control mice (brackets in Fig. 1d, k). Overall, these results suggested that BMT could recover the nuclear morphology and skin barrier function 2.5 months after IR injury.

### IR induced BMDC migration to the EDJ

We next investigated whether IR per se could induce the migration of BMDCs into the epidermis. To address this issue, only the head was protected by lead when the whole body of mice was exposed to IR (Fig. 2a). Thereafter, bone marrow cells from green fluorescent protein (GFP) transgenic mice in which GFP is ubiquitously expressed were injected via tail vein of the irradiated mice. We previously performed several experiments and found that tail vain injection is a suitable way to examine BMDCs in several organs, including the kidney, liver, and brain^12–14^. In this experiment, the head was considered as the control (the skin without IR exposure). Histological analyses revealed that GFP-positive BMDCs were detected in the skin 1 month after BMT, whereas they were not observed in the head skin (Fig. 2b, c). A high-power microscopic view showed that the epidermal GFP-positive cells had ramified processes (inset in Fig. 2c). BMDCs in the EDJ were then characterized using immunohistochemistry. GFP-positive cells, which represented BMDCs, were positive for several Langerhans cell (LC) markers, including MHCII, langerin, and EpCAM (Fig. 2d). Next, we examined the correlation between the BMDCs in EDJ and γδ-TCR, a marker of dendritic epidermal T cells in the epidermis, as a recent study showed that inflammation, followed by radiation-induced skin injury, is mediated by dendritic epidermal T cells^8^. We observed that BMDCs in EDJ were negative for γδ-TCR, suggesting that the BMDCs in EDJ were not mediators of radiation-induced inflammation (Fig. 2e). Quantitative analysis revealed the percentage of GFP^+^ MHCII^+^ cells in total MHCII^+^ cells in the epidermis of skin covered with or without lead protectors. While GFP^+^ cells were not observed in the epidermis of the head skin protected by lead, 11.4 ± 0.215% and 2.74 ± 0.159% MHCII^+^ cells were GFP positive in the body skin 10 weeks and 4 months after IR, respectively (Fig. 2f). GFP^+^ cells were no longer detected in the epidermis in 6 months after BMT (data not shown).

The fact that several LC markers are positive in BMDCs in EDJ shown in Fig. 2d led us to investigate these two types of cells further using BMT mice in which GFP-positive bone marrow cells were transplanted after 10 Gy IR exposure. As suggested by Fig. 2f, the population of BMDCs in EDJ was considerable 2.5 months after BMT, we analyzed those BMT mice, finding that BMDCs in EDJ and LCs appeared to be distinct from LCs in terms of their shape and spatial localization (Fig. 2g–l). Indeed, the morphological analysis showed that BMDCs extended several dendrites toward keratinocytes (Fig. 2g, i), whereas LCs extended fewer dendrites (Fig. 2j). In terms of localization, BMDCs were close to the EDJ (white dotted lines), whereas native LCs were located away from the junction (Fig. 2k, l). Quantitative analysis confirmed that BMDCs were predominantly located in the basal layers, whereas LC preferentially resided in the suprabasal layers, which included spinous, granular, and cornified layers lying above the basal layers of the epidermis (Fig. 2m, n,  *P* < 0.01).

To further distinguish between these two types of cells, we utilized the mechanical peeling technique. The epidermal cells were dissected into two populations; BMDCs, which were sorted based on the GFP^+^ CD45^+^ MHCII^+^ EpCAM^+^ population, and native LCs sorted based on the GFP^−^ CD45^+^ MHCII^+^ EpCAM^+^ population. We observed that BMDCs did not express inducible nitric oxide synthase (iNOS), unlike LCs^15^ (Fig. 2o,  *P*< 0.01). We next examined whether the cell population isolated using the mechanical peeling method might be contaminated with macrophages. To distinguish the characteristics of BMDCs from that of macrophages, we isolated two types of macrophages from the dermis. One was sorted as the GFP^+^ CD45^+^ MHCII^+^ EpCAM^+^ population (referred to as GFP^+^ Mϕ^1^), and the other as the GFP^+^ CD45^+^ F4/80^+^ CD11b^+^ population (referred to as GFP^+^ Mϕ^2^). The expression levels of several genes, which are good markers for macrophages, were compared. As shown in Fig. 2p, compared to LC and Mϕ, the BMDCs exhibited unique Mϕ marker expression patterns. In particular, Arginase 1 (Arg1) expression was found to be specific for BMDCs, while other markers were expressed only in macrophages (*P* < 0.01). These findings suggested that BMDCs were a unique population, which was distinct from Mϕ and LC.

### BMDCs induced keratinocyte proliferation for the recovery of radiation-induced skin injury

**Figure 3:**
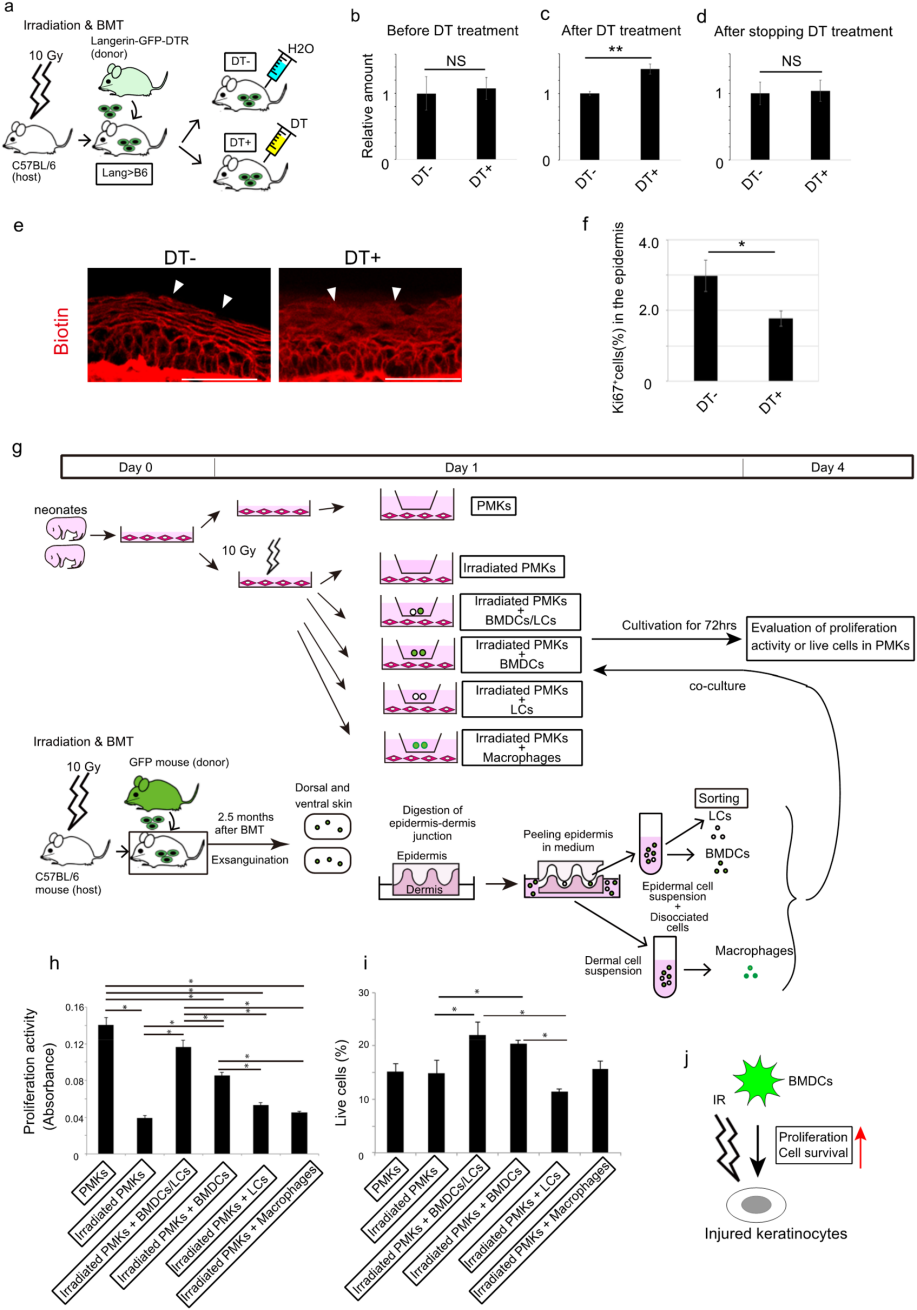
BMDCs act on irradiated keratinocytes. (**a**) Experimental design for detecting BMDCs in the epidermis. (**b**–**d**) TEWL was measured in DT^−^ and DT^+^ mice before and 1.5 months after DT or water treatments, and after stopping DT or water treatments. The average values of control mice were set to 1, and the bars show ± SE (n = 6). The experiments were repeated twice. **P < 0.01. (**e**) Representative images of biotin function assay of DT^−^ and DT^+^ mice are shown. Arrowheads indicate different biotin staining pattern in DT^−^ and DT^+^ mice. Scale bars, 50 µm. (**f**) One and a half months after DT or water treatments, the percentage of Ki67^+^ cells in the epidermis of the paw pad skin was significantly lower in DT^+^ mice than in DT^−^ mice. The average values indicate ± SE. Three mice were examined, and the experiments were repeated twice. *P < 0.05. (**g**) Schema of irradiated PMKs co-cultured with sorted cells from BMT mice. (**h**) Measurement of cell proliferation activity revealed that BMDCs, together with LC, most efficiently eliminated the suppressive effect of irradiation on cell proliferation. *P < 0.05. The experiments were repeated twice. (**i**) Percentage of live keratinocytes after co-culture with various combinations of cells revealed that BMDCs, together with LC, most efficiently made irradiated keratinocytes survive. *P < 0.05. (**j**) Summary of the result of in vitro experiments. PMKs, primary mouse keratinocytes; DT, DT, diphtheria toxin; BMDCs, bone marrow-derived cells.

The conditional deletion of epidermal BMDCs is a way of determining its specific role in the skin. For this purpose, recipient wild type mice receiving bone marrow cells from Langerin-GFP-diptheria toxin receptor (DTR) transgenic mice (Lang > B6) were generated (Fig. 3a). In this mouse model, we expected the administration of diptheria toxin (DT) to eliminate epidermal BMDCs expressing langerin promotor-associated DTR^16^. One month after BMT, Lang > B6 mice were divided into two groups. One group was treated with DT (referred to as DT^+^), and the other was treated with water (referred to as DT^−^). Using these mice, we performed a biotin function assay to visualize skin barrier conditions. A TEWL assay was also conducted to evaluate skin barrier function, as higher values of TEWL indicate skin barrier defects^17^. Before DT or water treatment, TEWL was confirmed to be identical between DT^−^ and DT^+^ mice (Fig. 3b). After 1.5 months, mice in the DT^+^ group showed significantly higher TEWL values than those in the DT^−^ group (Fig. 3c,  *P* < 0.01). In contrast, two weeks after stopping DT or water treatment, TEWL in the DT^+^ group returned to the same level as that in the DT group (Fig. 3d). To exclude the possibility that the effect of DT treatment was due to pharmacological side effects, we treated wild type mice with DT and measured TEWL after 1 month. No significant difference was observed between DT and water treatments (Supplemental Fig. 3). Consistently, biotin function assay also revealed that reticular biotin patterns observed in the epidermis of DT^-^ mice were significantly perturbed those in the epidermis of DT^+^ mice, which were similar to those 10 Gy irradiated mouse skin (Fig. 3e, Supplemental Fig. 4, compare those with Fig. 1f).

**Figure 4:**
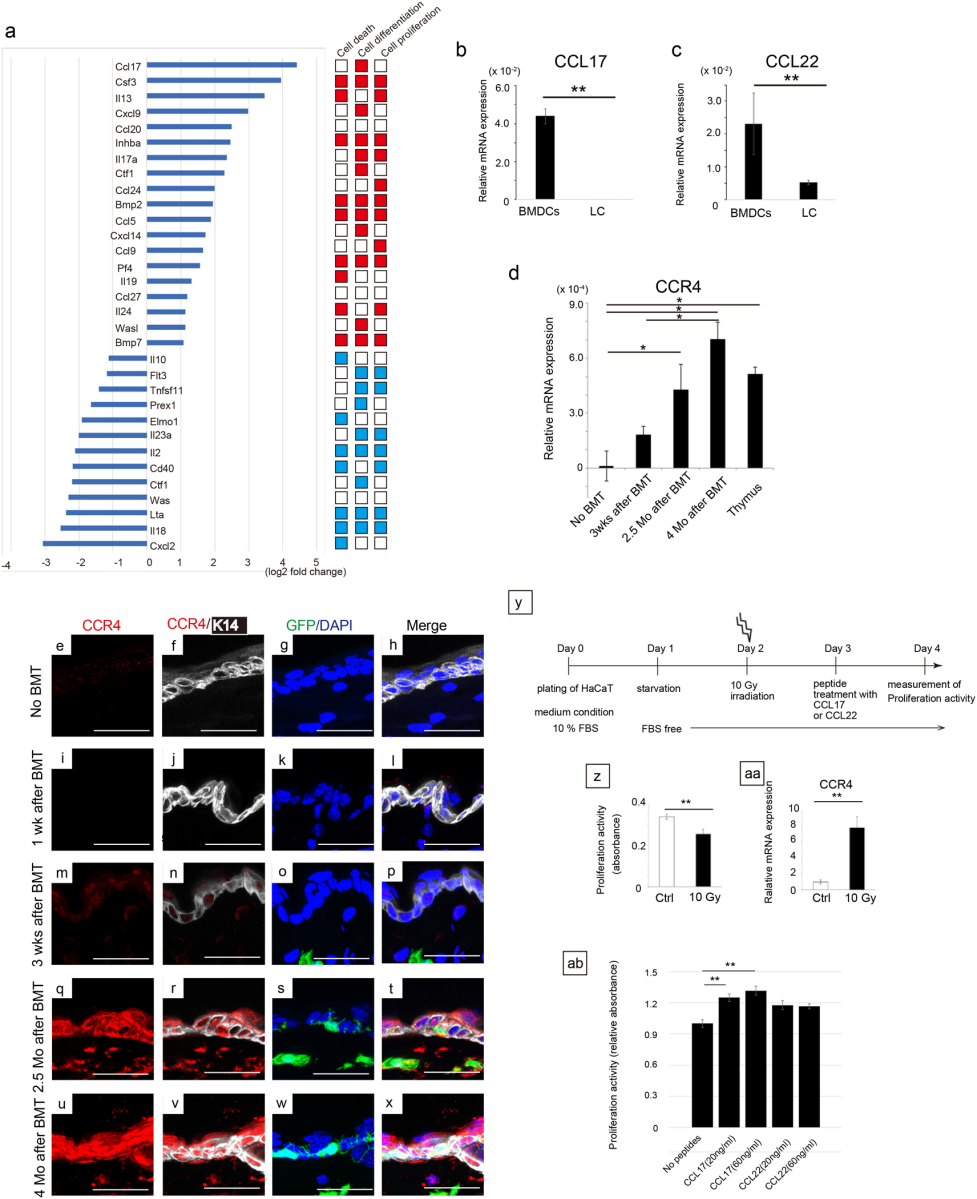
Crosstalk utilizing the CCL17-CCR4 axis between BMDCs and irradiated keratinocytes. (**a**) (Left panel) Partial results of microarray between BMDCs and LC are shown. (Right boxes) The function of each gene on cell death, differentiation, and proliferation reported on mouse genome informatics (http://www.informatics.jax.org/) is visualized in colors; red color indicates positive effects, while blue color indicates negative effects on each function. mRNA samples were collected from mice 2.5 months after BMT (n = 3, respectively). (**b**, **c**) CCL17 and CCL22 were significantly expressed in BMDCs, compared with LC. (**d**) CCR4 was induced in basal cells time-dependently after BMT. CCR4 expression in the thymus was compared as a positive control. wks; weeks, Mo; months. **P < 0.01, *P < 0.05. (**e**–**x**) Immunostaining with anti-CCR4 (red), anti-K14 (white; pseudo-color), GFP (green), and DAPI (blue) was performed using the skin of BMT mice at various time points. CCR4 was strongly expressed in the entire epidermal cells as well as in dermal cells 2.5 months and 4 months after BMT. Scale bars, 50 µm. (**y**) In vitro experimental protocol using the HaCaT cell line. (**z**) 10 Gy of IR suppressed proliferation activity of HaCaT. (**aa**) CCR4 was induced upon 10 Gy IR exposure in HaCaT on day 3. **P < 0.01. (**ab**) CCL17, but not CCL22, significantly recovered cell proliferation compared with no treated irradiated HaCaT. Experiments repeated twice. **P < 0.01. BMT, bone marrow transplantation; BMDCs, bone marrow-derived cells; LC; Langerhans cells.

The fact that the epidermis layer in BMT mice was significantly thicker than that in control mice (Fig. 1k) led to the hypothesis that the proliferation of basal cells could be involved in BMDC migration to the EDJ during skin barrier dysfunction recovery in BMT mice. Immunohistochemistry using anti-Ki67 antibody showed that the percentage of proliferative cells was significantly decreased in the epidermis of DT^+^ mice, suggesting that BMDCs might be involved in maintaining keratinocyte cell number, perhaps by stimulating keratinocyte proliferation (Fig. 3f,  *P* < 0.05, and Supplemental Fig. 5).

**Figure 5:**
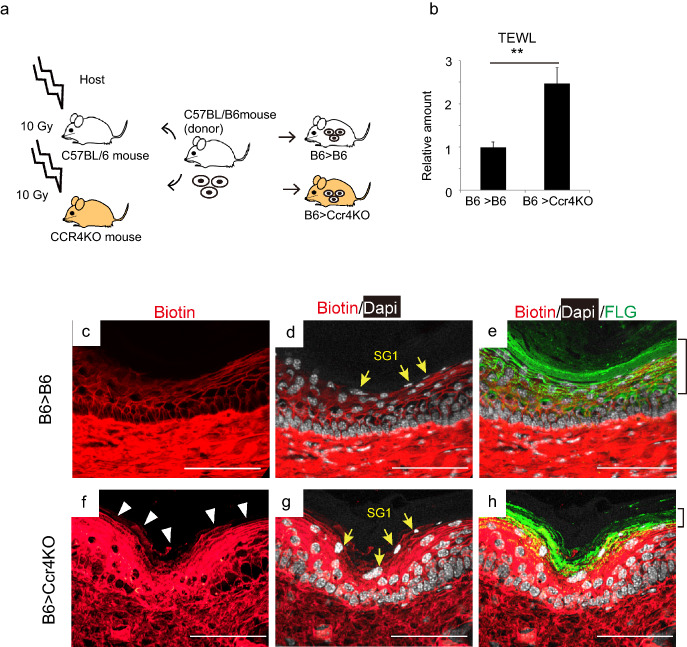
*Ccr4* deletion in the skin causes skin barrier dysfunction in mice. (**a**) Experimental design for CCR4-KO in the skin. (**b**) TEWL of B6 > *CCR4* KO significantly increased compared to that of B6 > B6 2.5 months after BMT. **P < 0.01. n = 6 for each. (**c**–**h**) Biotin function assay was performed 2.5 months after BMT. Reticular patterns of biotin-positive granular layers were observed in B6 > B6, while they were destroyed, and biotin stain surrounded SG1 in B6 > *CCR4* KO mice. Yellow arrows indicate SG1, while brackets show the filaggrin-positive layers. Scale bars, 50 µm. The experiments were repeated twice. TEWL, transepidermal water loss; BMT, bone marrow transplantation; KO, knockout; SG1, granular layer 1 of mouse skin.

Next, we assessed the role of BMDCs in keratinocyte proliferation using an in vitro system, in which irradiated keratinocytes were co-cultured with BMDCs, LCs, or Mϕ (Fig. 3g). First, primary mouse keratinocytes (PMKs) were isolated from neonatal mice^11^. After 24 h of cultivation, they were exposed to 10 Gy IR, followed by co-culture with sorted cells from BMT mice. After 4 days, IR-exposed PMKs exhibited lesser proliferative response than the control PMKs, suggesting that 10 Gy IR suppressed keratinocyte proliferation (Fig. 3h, comparison of “PMKs” with “Irradiated PMKs”, *P* < 0.05). In contrast, co-culture of irradiated PMKs with either BMDCs/LC or BMDCs alone most efficiently eliminated the suppressive effect of irradiation on cell proliferation (Fig. 3h “Irradiated PMKs”, ”Irradiated PMKs + BMDCs/LCs”, and “Irradiated PMKs + BMDCs”, *P* < 0.05). However, neither LC nor macrophages exerted significant effects on irradiated PMKs (Fig. 3h “Irradiated PMKs”, “Irradiated PMKs + LCs” and “Irradiated PMKs + Macrophages”, *P* < 0.05).

We also determined the percentage of living cells under each condition using an amine-reactive dye. We observed that the percentage of live keratinocytes co-cultured with BMDCs/LC or BMDCs was significantly higher than that in other groups (Fig. 3i “Irradiated PMKs”, “Irradiated PMKs + BMDCs/LCs”, and “Irradiated PMKs + BMDCs”). Overall, BMDCs recovered the damage in irradiated PMKs by promoting cell proliferation and increasing the percentage of live cells (Fig. 3j).

### BMDCs cross-talked with irradiated keratinocytes utilizing the CCL17-CCR4 axis

Microarray analysis (Supplemental data) showed that out of the 32 cytokines identified, 19 were significantly upregulated, whereas 13 were downregulated in BMDCs compared to in LCs obtained from mice 2.5 months after BMT (Fig. 4a). We were interested in CCL17, as it was most abundantly expressed in BMDCs compared to in LC (Fig. 4a). Subsequently, real-time PCR analysis showed that CCL17 was abundantly expressed in BMDCs, but almost not expressed in LC (Fig. 4b,  *P* < 0.01). As CCL22 is another ligand of CCR4^18^, we assessed CCL22 expression and found it to be expressed significantly in BMDCs compared to in LC (Fig. 4c,  *P* < 0.01). We sorted basal cells as CD45^−^ MHCII^−^ integrinα6^−^ Sca1^−^ population^10,11^ to examine CCR4 expression, as stem cells reside in the basal cell layers with proliferative potential^19^. CCR4 expression was not detected in basal cells under control conditions, while IR significantly induced CCR4 expression in the basal cells of BMT mice (Fig. 4d,  *P* < 0.05). It is noteworthy that 2.5 or 4 months after IR exposure, CCR4 expression was as high as that in the thymus, which is known to express CCR4 abundantly (Fig. 4d). IR also induced CCR4 in PMKs, which was used to establish BMDC co-culture (Fig. 3, Supplemental Fig. 6). As CCL17 secreted by basal cells may act on themselves in an autocrine manner, we investigated its expression in sorted basal cells from BMT mice; however, we did not detect CCL17 expression (Supplemental Fig. 7), suggesting that CCL17 did not act on basal cells in an autocrine fashion.

**Figure 6:**
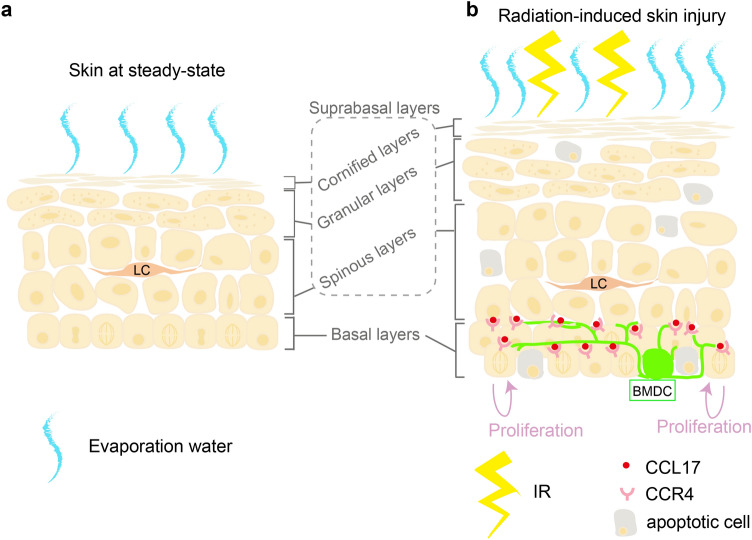
Summary of the study. (**a**) The skin at steady state. (**b**) In response to IR, BMDCs migrate to the injured skin. CCL17 secreted by BMDCs binds to CCR4, which is induced in epidermal cells to stimulate cell proliferation and recover from radiation-induced skin injury. As a result, the epidermis becomes thicker than that at the steady state. IR, ionising radiation; BMDCs, bone marrow-derived cells; LC; Langerhans cells.

Next, we investigated the expression of CCR4 using immunohistochemistry and observed that CCR4 was not detectable in mouse skin either before or 1 week after BMT (Fig. 4e–l). Conversely, CCR4 was weakly induced in all epidermal layers 3 weeks after irradiation and strongly induced even in dermal cells after 2.5 and 4 months after irradiation/BMT (Fig. 4m–x). We did not observe any signal in sections in which hamster IgG for the negative control was applied instead of the hamster anti-CCR4 antibody (Supplemental Fig. 8).

Finally, using an in vitro system, we attempted to determine the relative contribution of CCL17-CCR4 versus CCL22-CCR4 to the recovery process after radiation-induced skin injury. Although we first utilized PMKs, these cells were too fragile to endure constant experimentation under starvation and exposure to 10 Gy IR; hence, we decided to use a commonly used human keratinocyte cell line, HaCaT (Fig. 4y). Irradiation with 10 Gy significantly suppressed the proliferation of HaCaT cells (Fig. 4z,  *P* < 0.01). Conversely, consistent with the result obtained using mouse skin, CCR4 expression was significantly induced upon 10 Gy irradiation in irradiated HaCaT cells 1 day after IR exposure (Fig. 4aa). CCL17 or CCL22 peptide treatment revealed that CCL17, but not CCL22, significantly recovered the proliferation of irradiated HaCaT cells, suggesting that CCL17 was the key molecule involved in recovery from radiation injury (Fig. 4ab,  *P* < 0.01).

### BMDCs failed to recover the skin barrier function after IR-induced injury in mice with CCR4-deficient keratinocytes

Finally, to assess the role of CCR4 in our model, BMT from either wild type mice (B6 > B6) or *Ccr4* knockout mice (B6 > *Ccr4* KO) to wild type mice was performed (Fig. 5a). TEWL was significantly higher in B6 > *Ccr4* KO mice than in B6 > B6 mice 2.5 months after BMT (Fig. 5b,  *P* < 0.01). In addition, biotin function assays revealed that the biotin-stained reticular patterns were destroyed in the SG1 layers of B6 > *Ccr4* KO (Fig. 5f–h) but maintained in the epidermis of B6 > B6 skin (Fig. 5c–e). In addition, the nucleus of keratinocytes in the SG1 layer showed oval shape in the skin of B6 > *Ccr4* KO mice, while those cells of the corresponding layer were spindle-like in B6 > B6 mice (Fig. 5d, g). The filaggrin-positive layers appeared to be thinner in B6 > *Ccr4* KO mice than in B6 > B6 mice (brackets in Fig. 5 e, h). Altogether, barrier dysfunction due to IR was not recovered in B6 > *Ccr4* KO mice.

## Discussion

This study focused on the functional role of BMDC migration into the EDJ of the epidermis as a sequel to our previous study, where we reported this phenomenon. We demonstrated that whole-body IR exposure impaired skin barrier function in 7 days; however, BMDCs from healthy donor mice recovered the skin barrier dysfunction 2.5 months after BMT. The recovery process involved the accelerated proliferation of basal cells in response to significant apoptosis of epidermal cells after IR exposure. Notably, IR exposure led to BMDC migration to the EDJ, from which CCL17 was released to bind to CCR4 on basal cells, resulting in keratinocyte proliferation (Fig. 6).

Although the BMDCs express several markers that are shared with LCs, these two cell types are distinct in the radiation-injured skin with respect to the following: (i) morphologically, BMDCs appeared to extend more abundant dendritic processes toward keratinocytes than LCs, (ii) anatomically, BMDCs were located in EDJ where LCs were not observed; (iii) at the molecular level, the gene profile of BMDCs was distinct from those of LCs. In fact, the functions of LCs and BMDCs differ considerably; usually, LCs are observed in the suprabasal layers of the epidermis where they incorporate microbial antigens^20^, whereas BMDCs are temporary cells that migrate into the radiation-injured skin prone to apoptosis and assist in the proliferation of the basal cells as demonstrated in the present study. Until now, our research group has focused on the roles of BMDCs, especially in damaged peripheral nerves, and observed that BMDCs promote nerve regeneration or relieve pain^21,22^. Interestingly, BMDC migration in the damaged nerve is spatiotemporal, as observed in our previous study; BMDCs migrate and release brain-derived neurotrophic factor to not the proximal, but the distal end of the damaged sciatic nerve until 4 weeks after the crash, accompanied by nerve fiber regeneration 2.5 months after BMT^22^. Notably, the time required for recovery from the damaged nerve was similar to that required for the recovery from skin barrier defects in the present study, while the key molecule for the crosstalk with damaged epidermal cells is unique to the skin.

In elucidating the key molecule used crosstalk between BMDCs and damaged epidermal cells, we hypothesized that soluble molecule(s) might be released from the extended ramified processes of BMDCs toward damaged keratinocytes (basal and suprabasal cells) in a paracrine manner. Hence, we focused on CCL17, the chemokine of which was identified in microarray to be the most abundantly expressed in BMDCs compared to that in LCs. CCL17 stimulated the proliferation of basal cells after apoptosis due to IR exposure. Furthermore, we found that CCR4, the receptor of CCL17, was expressed in the basal cells 3 weeks after IR exposure, before BMDC migration to the EDJ. This is probably because of IR-induced inflammation and apoptosis in epidermal cells^5^. Consistently, CCR4 expression is also induced in epidermal cells by allergic skin inflammation, such as atopic dermatitis^5,23^.

The potent mitogenic action of CCL17 in the basal cell was also compatible with the study using Grainyhead-like 3 *(Grhl-3)* knockout mice^24^. The absence of GRHL3, which is normally expressed in suprabasal cells, leads to barrier dysfunction in the skin, followed by CCL17 secretion from suprabasal cells as well as basal cells. Excessive expression of CCL17 in the entire epidermal cells (suprabasal/basal cells) accelerates proliferation of the basal cells, accompanied with the accumulation of immune cells, resulting in skin barrier dysfunction in *Grhl-3* knockout mice. In contrast, our study showed the positive effect of CCL17 to recover skin barrier defects from radiation-induced injury. These inconsistent results regarding barrier dysfunction recovery might be due to the difference in CCL17 concentration. Excess CCL17 mediates severe inflammation in atopic dermatitis and osteoarthritis^23,25^. This might be the case with *Grhl-3* knockout mice. Conversely, irradiated epidermal cells per se were unable to express CCL17 (Supplemental Fig. 7), probably because of radiation-induced skin injury. Instead, the BMDCs that migrated to the EDJ ramified toward the basal cells to secrete CCL17 and promote proliferation and the population of BMDCs in the EDJ was thinly scattered compared to the total number of epidermal cells. Therefore, it is plausible to expect that CCL17 concentration is lower in irradiated skin than in *Grhl-3* knockout mice. As a result, skin barrier dysfunction was recovered.

Our results showed that CCL22, another ligand of CCR4, did not affect the proliferation of basal cells. Notably, CCL22 was expressed in both LCs and BMDCs. As LCs express CCL22 under specific chronic inflammatory conditions, CCL22 secreted by BMDCs appears the occurrence of chronic inflammation, which is a characteristic of radiation-caused dermatitis^2,26^. The function of CCL22 in radiation-caused dermatitis should be investigated in the future.

This study has several limitations, one of which is the mechanical peeling method used to isolate target BMDCs located in EDJ^5^. We have previously demonstrated that this method is efficient in dissociating most of the BMDCs from EDJ while preparing epidermal sheets^5^. A major concern for this method is the contamination with macrophages, as BMDCs and macrophages share several cell surface markers (GFP^+^ CD45^+^ MHCII^+^ EpCAM^+^). To address this issue, additional quantitative PCR was performed to identify their unique gene expression patterns.

In conclusion, we determined the molecular and functional roles of BMDCs in repairing radiation-induced skin damage. This is the first study to show that BMDCs play an important role in the repair of irradiated skin. As BMDCs are capable of stimulating the recovery of the epidermis from IR-induced injury, it is conceivable that the therapeutic aspects of BMDCs can be applied to radiation-induced skin ulcers, which are often intractable to surgical treatments in the clinical setting. Thus, further investigations for determining the role of BM-cells in skin diseases are required for developing novel therapies.

## Methods

### Ethics

All animal experiments were carried out in compliance with the guidelines of Shiga University of Medical Science for the care and use of animal research and the ARRIVE guidelines (Animal Research: Reporting of In Vivo Experiments). The protocol was approved by the Committee on the Ethics of Animal Experiments of Shiga University of Medical Science (Permission Number: #2017-2-6).

### Mice

Eight- to ten-week-old C57BL/6J mice were purchased from CLEA Japan, Inc. (Osaka, Japan). C57BL/6-Tg (UBC-GFP) and 30Scha/J (GFP-Tg) mice were purchased from The Jackson Laboratory (Bar Harbor, ME, USA). Langerin-diphtheria toxin receptor (DTR) mice were gifted by Prof Kabashima of Kyoto University after the approval of the founder, Dr Bernard Malissen^27^. B6;129P-Ccr4^tm1pwr^/J mice were obtained from Dr Nakamura’s laboratory^23^. Mice were genotyped using polymerase chain reaction (PCR) with tail DNA and the primers shown in Supplementary Table 1. For the in vivo experiments, mice were anesthetized with 1.5% isoflurane (Wako Co., Tokyo, Japan) using an inhalation gas anesthesia system for small laboratory animals.

### Bone marrow transplantation

Bone marrow cells were isolated from 6–8-week-old C57BL/6 J, GFP-Tg, or langerin-DTR mice. The recipient mice were irradiated with 10 Gy X-irradiation in single fraction. 4 × 10^6^ bone marrow cells from the donor were injected into the tail vein of the recipient mice immediately after irradiation. For the protector model, the heads of the mice were protected with 5-mm thick lead protectors when radiation was applied. While the intestine is radio-sensitive, mice can survive longer without any symptom of gastrointestine after BMT^28^.

### Diptheria toxin (DT) treatment

One month after BMT from Langerin-GFP-DTR mice, 1 µg diphtheria toxin (DT) (Wako)/200 µL phosphate-buffered saline (PBS) was injected intraperitoneally every four days for a total of 11 times to eliminate BMDCs continuously in the epidermis, as langerin-positive cells are known to return four weeks after a single DT treatment^29^. After 1.5 months of DT treatment, transepidermal water loss **(**TEWL) measurements or biotin function assays were performed.

### Immunohistochemistry

After exsanguination, pieces of skin were fixed with 4% paraformaldehyde overnight and embedded in Tissue-Tek OCT compound (Sakura Finetek Japan Co., Tokyo, Japan). For immunohistochemistry, the sections (10 µm) were treated with 0.05% goat serum and 0.1% foetal bovine serum in PBS for 1 h at 25 °C and incubated with primary antibodies overnight at 4 °C, followed by incubation with secondary antibodies for 2 h at 25 °C. To detect the chemokine receptor (CCR) 4 signal, we performed antigen retrieval using HistoVT one (Nacalai Co., Kyoto, Japan), followed by treatment with the following primary antibodies: anti-fillagrin (1:400, Biolegend, San Diego, CA, USA), anti-keratin 14 (1:400, Biolegend), anti-MHCII biotin (1:200, eBioscience, San Diego, CA, USA), anti-TCR (1:50, Santa Cruz Biotechnology, Dallas, TX, USA), anti-CCR4 (0.5 mg/mL, Nagakubo lab), and anti-Ki67 (1:100, Abcam, Boston, MA, USA). The secondary antibodies used were Alexa Fluor 488, Alexa Fluor 555, Alexa Fluor 647, goat anti-rabbit, goat anti-guinea pig, and goat anti-hamster IgG (1:1000, Life Technologies, California, CA, USA). To detect Biotin, Biotium Streptavidin, we used CF555 (1:400, Thermo Fisher Scientific, Waltham, MA, USA). Sections for immunofluorescence were mounted with Vector Shield using 4′-6-diamidino-2-phenylindole (DAPI) (Vector Laboratories, Burlingame, CA, USA) and photographed using a laser scanning confocal microscope (EZ-C1, Nikon Co., Tokyo, Japan). The number of GFP- and/or MHCII-positive cells in the epidermis was quantified in each 3^rd^ section of the skin with or without lead protectors (20 sections per sample). Three mice were examined for each of with or without lead protectors. The distribution of BMDCs and LC in the skin layers was examined in 90 cells. Three mice were examined. To analyse proliferative epidermal cells, we quantified the numbers of Ki67-positive cells in epidermal cells in the wild type mice receiving bone marrow cells from langerin-DTR mice treated with DT or water (20 sections per sample); three mice were examined per group.

### Biotin function assays

Biotin function assays were performed as described previously^30^. Briefly, 10 mg/mL EZ-Link Sulfo-NHS-LC Biotin (Pierce Chemical Co., Dallas, TX, USA) in PBS was subcutaneously injected into the plantar region. After 30 min of incubation, the skin was isolated to establish frozen sections. The staining protocol is described above.

### TEWL measurement

Mice were anaesthetised via 1.5% isoflurane inhalation, after which, the hair on the dorsal skin was shaved with an electric clipper and removed using hair removal cream. The next day, TEWL was determined using a Vaposcan (Asahi Techno Lab. Co., Kanagawa, Japan) five times in the same back region per mouse, and the median values were obtained. The data were presented by setting control values to 1.

### Flow cytometry

The preparation of the epidermal cell suspension and collection of dissociated cells were performed as described previously^5^. Briefly, after exsanguination, the split ventral and dorsal skin of the ear was incubated in 0.5% dispase (Roche, Bale, Switzerland) for 45 min at 37 °C. Alternatively, trunk skin sheets were obtained after exsanguination and incubated overnight with 0.25% trypsin (Wako) at 4 °C. After separation of the epidermis from the dermis, the epidermal sheets were incubated in 0.3% trypsin containing 0.1% DNase (Sigma, St. Louis, MO, USA) for 10 min at 37 °C. Thereafter, it was shaken to release the epidermal cells. Then, the same volume of Roswell Park Memorial Institute (RPMI) 1640 medium containing 10% FBS was added, inverted gently 20 times, and filtered to obtain the epidermal cell suspension. A cluster of dissociated cells from the EDJ was salvaged using the RPMI medium, followed by the addition of 0.1% DNase and centrifugation at 1,400 rpm for 10 min at 4 °C. The dissociated cells were filtered to remove debris and combined into an epidermal cell suspension. An amine-reactive dye (LIVE/DEAD fixable violet dead cell staining kit (Life Technologies)) was routinely used to detect dead cells and anti-mouse CD16/32 antibody (10 µg/mL) (clone 2.4G2, BD Pharmingen, Franklin Lakes, NJ, USA) was used to block Fcγ receptors before staining. The antibodies used for sorting included anti-CD45 (30-F11, BD Pharmingen), anti-MHCII (clone M5/114.15.2, eBioscience), anti-Sca-1 (D7, BD Pharmingen), and anti-Integrin α6 (GoH3, eBioscience). The CD45^−^ MHCII^−^ cells were sorted to collect keratinocytes, while CD45^−^ MHCII^−^ Sca-1^+^ integrin α6^+^ cells were sorted to collect the basal cells^11^. The anti-annexin V antibody (BD Pharmingen) was used to detect apoptotic cells, and 1.0 × 10^6^ cells in suspension from control or BMT mice were sorted to compare the percentage of amine-reactive dye^+^ annexin V^+^ cells in CD45^−^MHCII^−^ cells. For sorting BMDCs, anti-EpCAM (G8.8, eBioscience) antibodies, as well as anti-CD45 and anti-MHCII antibodies, were used. To sort macrophages, we prepared a dermal cell suspension. Briefly, the dermis, after removing the dissociated cells from the epidermis, was minced and incubated in RPMI medium with 0.1% DNase containing 100U/mL collagenase IV (Worthington Biochemical Corporation, Lakewood, NJ, USA) for 90 min at 37 °C. Then, it was pipetted 20 times and filtered twice. After blocking with the anti-mouse CD16/32 antibody, anti-CD45, anti-F4/80 (BM8, BioLegend), and anti-CD11b (M1/70, BioLegend) antibodies were used to sort macrophages in the dermal cell suspension. Another set of anti-CD45, anti-MHCII, and anti-EpCAM antibodies were used to sort macrophages in the dermal cell suspension. Data were collected using FACSAria Fusion (BD Biosciences, San Jose, CA, USA).

### RNA isolation and cDNA synthesis

RNeasy micro kit (Qiagen, Valencia, CA, USA) was used for RNA isolation from BMDCs and Langerhans cells (LCs), followed by the Ovation Pico WTA system (Nugen, Redwood City, CA, USA) to generate cDNA. For RNA isolation from cultured cells, an RNeasy micro kit was used, followed by cDNA synthesis using Takara Primescript RT reagent kit with gDNA eraser (Takara Bio Co., Shiga, Japan).

### Real-time PCR

Real-time PCR was performed in triplicate using Light Cycler 480 SYBR Green I master mix (Roche, Mannheim, Germany). All reactions were performed in a 10 µl reaction volume and the mRNA expression levels were determined using the 2^−ΔCT^ method. Gene expression was normalised to that of the genes encoding ribosomal protein large P0 (RPLP0) or actin. The primer sequences are shown in Supplementary Table 2.

### Microarray

Microarray analysis, including data processing, was performed by Takara Bio Co. using independent samples of BMDCs and LCs from three mice 2.5 months after BMT, respectively.

### Primary keratinocyte culture and co-culture

Keratinocytes for primary keratinocyte culture were prepared as previously described^11^. Briefly, the neonate skin was unwrapped from the body using forceps after CO_2_ exposure and decapitation^31^. Then, the skin was incubated in 0.5% dispase overnight at 4 °C. On the following day, the epidermis was separated from the dermis and incubated with TrypLE (Invitrogen, Carlsbad, CA, USA) for 10 min, shaking at 37 °C. Then, CnT-PR (CELLnTEC, Bern, Switzerland) was added and pipetted 20 times to create the epidermal cell suspension. After filtration and centrifugation at 200×g for 10 min, 1.5 × 10^4^ keratinocytes were plated per well in rat tail type I collagen (Sigma)-coated 48-well plates (Corning Life Sciences, New York, NY, USA) without any feeder layer. After 1 day of incubation, the keratinocytes were exposed to 10 Gy IR, followed by co-culture with 5 × 10^3^ dendritic cells in a cell culture insert (Corning Life Sciences). CnT-PR was used for cultivation without adding serum because this medium included serum. The ratio of keratinocytes to LC was determined with reference to that of normal skin^32^. Three days after co-culture, cell proliferation activities were measured, or keratinocytes were sorted to evaluate live cells.

### HaCaT culture and treatment with CCL17 and/or CCL22 peptides

HaCaT keratinocytes (a kind gift from Dr Takaishi, Kochi University, Japan) were cultured in Dulbecco’s modified Eagle’s medium (Wako) supplemented with 10% foetal calf serum (Wako), 1% penicillin/streptomycin (Sigma), and 25 mM HEPES (Nacalai) at 37 °C in the presence of 5% CO_2_. They were starved and irradiated with 10 Gy IR, followed by treatment with human CCL17 and/or CCL22 (R&D system Minneapolis, USA), as described in the results sections with the aim of each experiment. The concentration of CCL17 or CCL22 were either 20 ng/ml or 60 ng/ml.

### Proliferation assay

At the end of cultivation, WST-1 (Takara Bio Co.) was added according to the manufacturer’s instructions, and the absorbance at 440 nm was measured using a multi-well plate reader (Infinite F200, TECAN, Kawasaki, Japan). The absorbance at 650 nm was used as the reference wavelength. Experiments were performed twice, and representative data are shown.

### Statistical analyses

Data were evaluated using t-tests to analyse differences between two groups or using one-way ANOVA to analyse differences among more than the three groups. Data were expressed as mean ± SE. *P* < 0.05 was considered significant.

## Supplementary Information

Supplementary Figures.

Supplementary Tables.

Supplementary Information.
