# Supplementary material for: A novel role for bone marrow-derived cells to recover damaged keratinocytes from radiation-induced injury
Source: Sci Rep. 2021 Mar 11;11:5653. doi: 10.1038/s41598-021-84818-1 (PMC7952382; doi:10.1038/s41598-021-84818-1)
Supplement: Supplementary file 1 — Supplementary Figures. [file 41598_2021_84818_MOESM1_ESM.pdf]

A novel role for bone marrow-derived cells to recover damaged keratinocytes from radiation-induced injury

Junko Okano[1, *], Yuki Nakae[2], Takahiko Nakagawa[3], Miwako Katagi[2], Tomoya Terashima[2], Daisuke Nagakubo[4], Takashi Nakayama[5], Osamu Yoshie[6], Yoshihisa Suzuki[1] and Hideto Kojima[2]

[1]Department of Plastic and Reconstructive Surgery, [2]Department of Stem Cell Biology and Regenerative Medicine, Shiga University of Medical Science, Shiga, [3]Department of Nephrology, Otowa Hospital, Kyoto, [4] Division of Health and Hygienic Sciences, Faculty of Pharmaceutical Sciences, Himeji Dokkyo University, Hyogo, Hyogo, [5]Division of Chemotherapy, Kindai University Faculty of Pharmacy, Osaka, [6] The Health and Kampo Institute, Miyagi, Japan

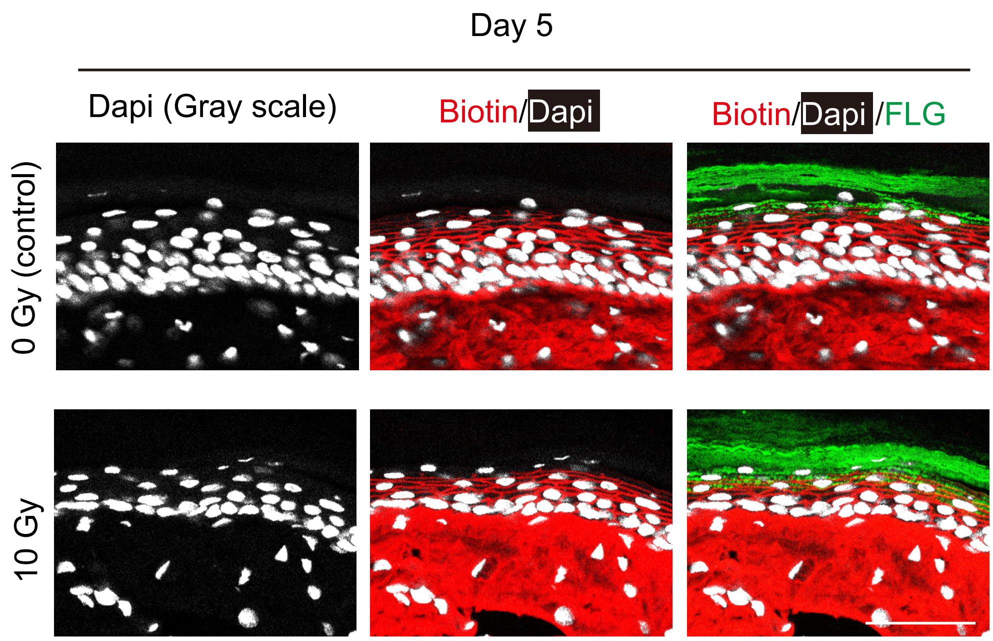

Supplemental Fig. 1

Representative skin images 5 days after control or 10 Gy radiation show no obvious skin barrier defects in the skin exposed to 10 Gy of IR. Scale bars, 20 μm.

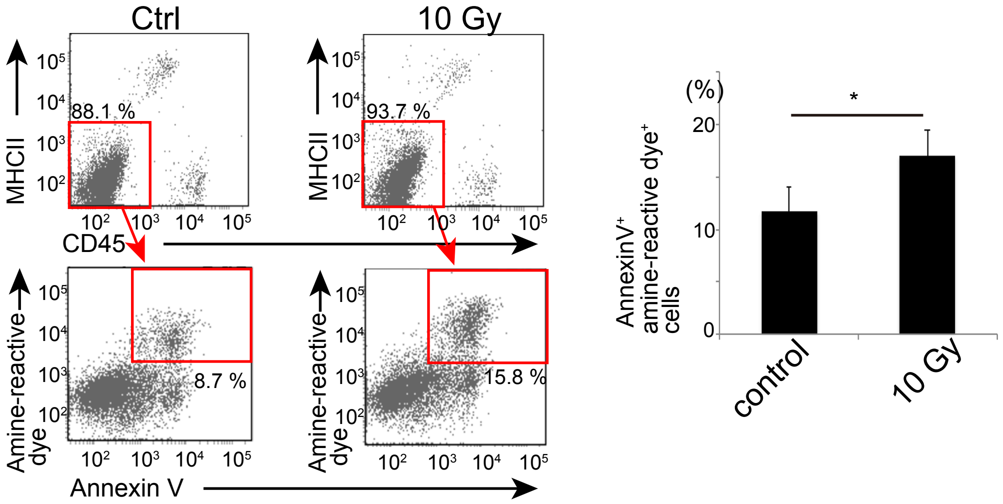

Supplemental Fig. 2

Apoptosis of epidermal cells was significantly increased in mice 7 days after 10 Gy IR exposure. $P < 0.05$. Three mice in each group were examined, and experiments were repeated twice.

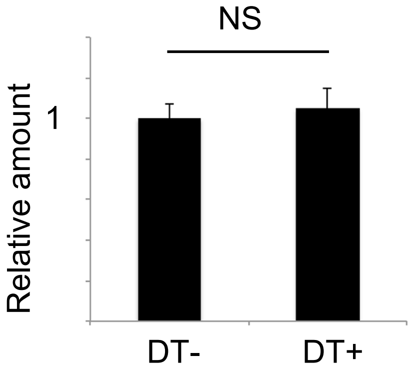

Supplemental Fig. 3

TEWL measurements of DT- or water-treated (DT+ or DT-, respectively) B6 mice showed no significant difference between the two groups. The average values of DT- mice are set as 1, and bars show ± SE (n=4). Experiments were repeated twice. **P < 0.01. DT, diphtheria toxin; TEWL, transepidermal water loss.

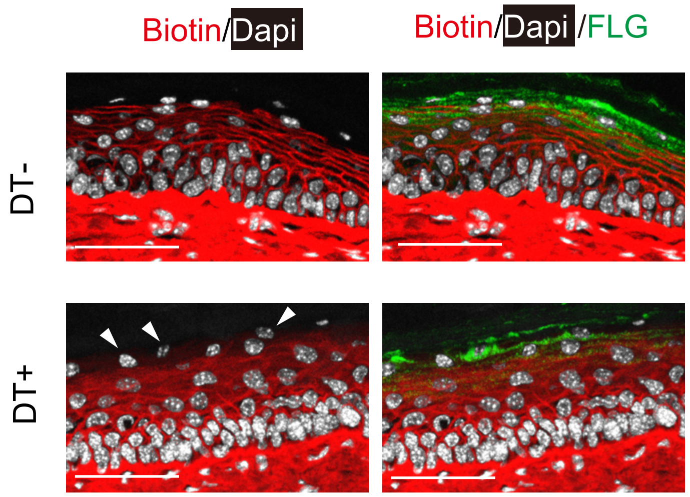

Supplemental Fig. 4

Biotin assay revealed skin barrier defects in DT-treated Lang>B6 mice. The nucleus of keratinocytes in SG1 layer (arrowheads) are rounded as observed in 10 Gy-exposed skin (compare them with Fig. 1e). Scale bars; 50 μm. DT, diphtheria toxin; SG, granular layer 1 of mouse skin.

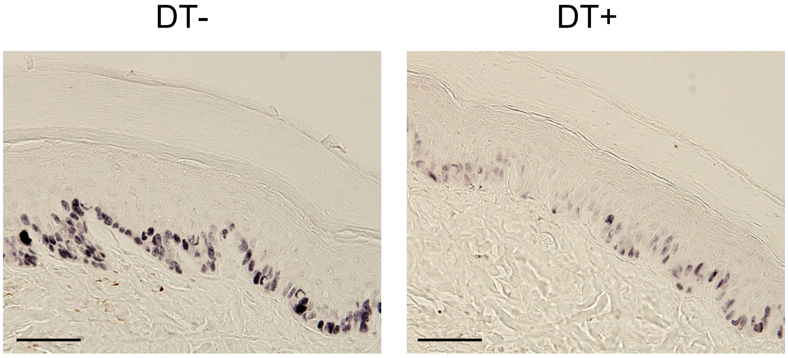

Supplemental Fig 5

Representative immunohistochemistry images of Ki67 of plantar skin of DT- and DT+ Lang>B6 mice. Scale bars; 20 μm. DT, diphtheria toxin.

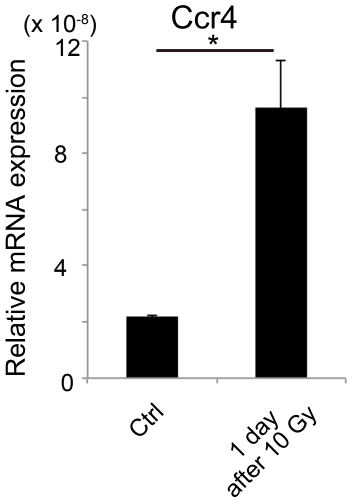

Supplemental Fig. 6

Ccr4 expression is induced in PMKs in vitro by IR. Experiments were repeated twice. *P < 0.01.

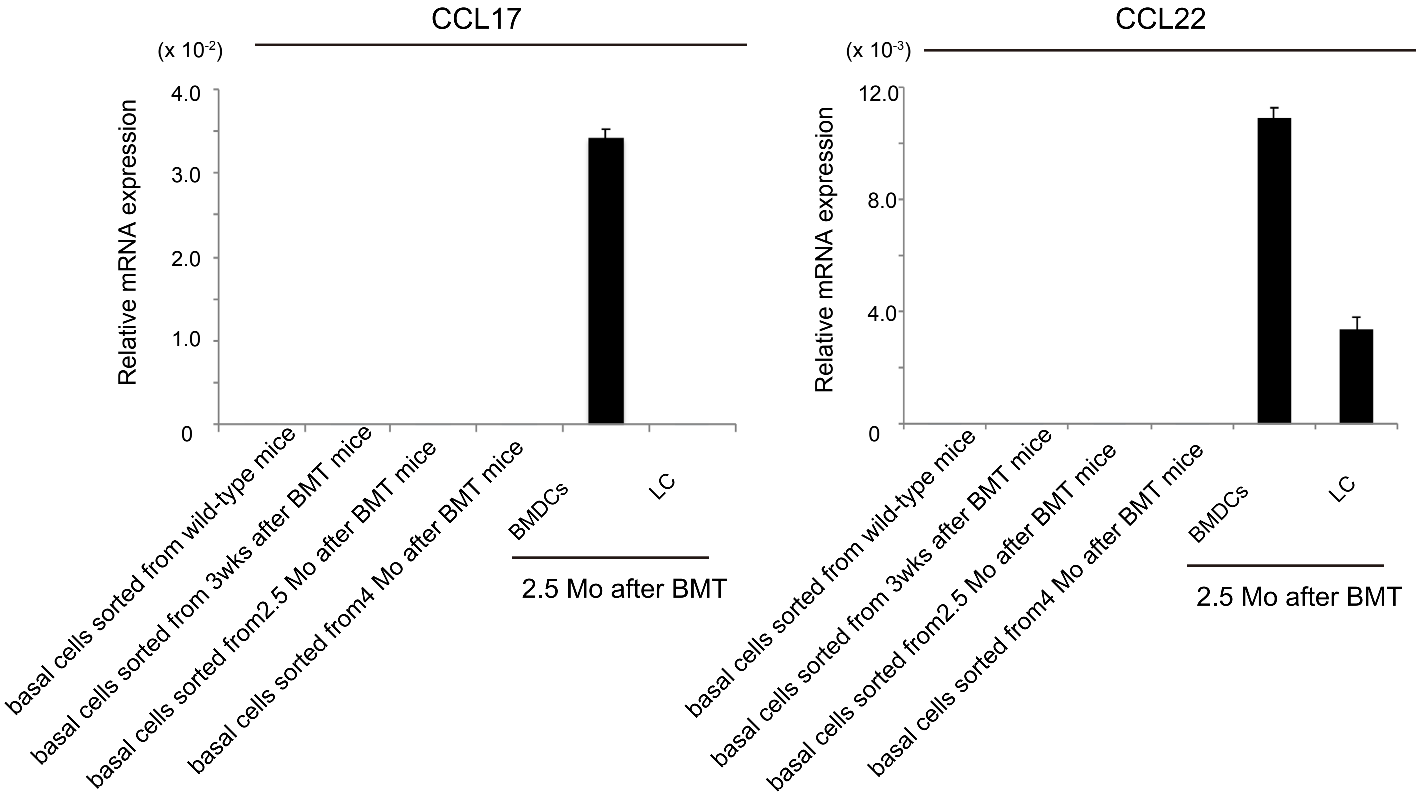

Supplemental Fig. 7

Neither CCL17 nor CCL22 expression was not detected in sorted basal cells from wild type or BMT mice, whereas abundant expression of both molecules was observed in BMDCs. Experiments were repeated twice. BMT, bone marrow transplantation; BMDCs, bone marrow-derived cells.

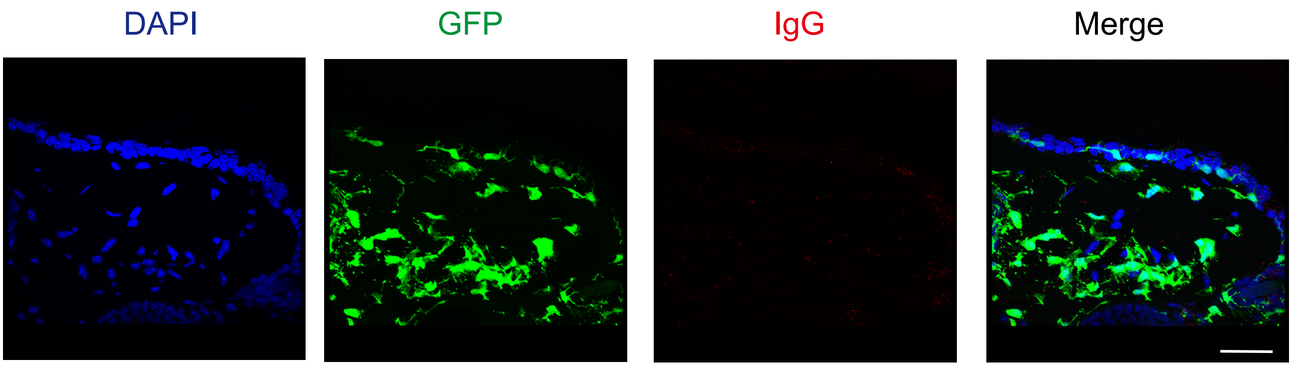

Supplemental Fig. 8

No specific signal was detected in sections in which hamster IgG was applied instead of hamster anti-CCR4 antibody for negative control. Scale bars, 20 μm.
