# Supplementary material for: A novel role for bone marrow-derived cells to recover damaged keratinocytes from radiation-induced injury
Source: Sci Rep. 2021 Mar 11;11:5653. doi: 10.1038/s41598-021-84818-1 (PMC7952382; doi:10.1038/s41598-021-84818-1)
Supplement: Supplementary file 2 — Supplementary Tables. [file 41598_2021_84818_MOESM2_ESM.pdf]

Supplementary table 1

Genotyping of GFP-Tg, Langerin-DTR mice or B6.

Mouse line Target product	Forward primer	Reverse primer
GFP-Tg	GCACCATCTTCTTCAAGGAC	ACTTGTACAGCTCGTCCATG
Product size: 420 bp		
Cycling conditions: 95°C for 15 min, 37 cycles of 94°C for 30 s, 55°C for 30 s and 72°C for 40 s followed by 72°C for 1 min		
Langerin-DTR-GFP		
Langerin-EGF	CACAGCCTGTGGTCTTTCTGTCTT<br>CAAGTCCGCCATGCCCGAAGGCTAC	GTAGCTTTTATATGGTCAGCCAAGG
Product size: 750 bp		
Cycling conditions: 97°C for 2 min, 35 cycles of 97°C for 30 s, 68°C for 30 s and 72°C for 1 min followed by 72°C for 5 min		
HBEGF	GGGACCCATGTCTTCGGAAAG	TTTTCCACTGGGAGGCT
Product size: 180 bp		
Cycling conditions: 94°C for 3 min, 35 cycles of 95°C for 15 s, 55°C for 15 s and 72°C for 20 s followed by 72°C for 5 min		
B6. 129P-Ccr4^{tm1pwr}		
CCR4	CCAAAGATGAATGCCACAGAGGTCACAG	TTACAAAGCGTCACGGAAGTCATG
Product size: 1291 bp		
CCR4KO	CCGGTTCTTTTTGTCAAGACCG	CGGCAGGCGCAAGGTGAGAT
Product size: 199 bp		
Cycling conditions: 94°C for 3 min, 35 cycles of 95°C for 30 s, 60°C for 30 s and 72°C for 30 s followed by 72°C for 2 min		

Supplementary table 2

Primer sequences used in this study.

Gene	Forward	Reverse
iNOS	GGCACCGAGATTGGAGTTC	TATGGAGCACAGCCACATTG
Arg1	AGCACTGAGGAAAGCTGGTC	CCATTCTTCTGGACCTCTGC
MR	CCCATTTATCATTCCCTCAGC	GGTTCCATCACTCCACTCAAAG
Retnla	ATCGTGGAGAATAAGGTCAAGG	CGAGTAAGCACAGGCAGTTG
Ym1	TATGCCTTTGCTGGAATGC	TGACGGTTCTGAGGAGTAGAGAC
CCL17	AGTGGAGTGTTCCAGGGATG	CAGGGACTTCTGCTCTGTGG
CCL22	CCAGGACTACATCCGTCACC	TGGCAGAAGAATAGGGCTTG
CCR4 (mouse)	GCATTGCTTCATAGACTGTCCTC	TGCCTTGATACCTTCCTTGG
RPLP0	ATCAATGGGTACAAGCGCGTC	CAGATGGATCAGCCAGGAAGG
CCR4 (human)	GCCTCACAGACCTTCCTCAG	AACAGGACCAGAACCACCAC
Actin (human)	GGACTTCGAGCAAGAGATGG	AGCACTGTGTTGGCGTACAG
